# Dynamics of confined water inside carbon nanotubes based on studying tetrahedral order parameters

**DOI:** 10.1038/s41598-024-66317-1

**Published:** 2024-07-05

**Authors:** Amit Srivastava, Sufian Abedrabbo, Jamal Hassan, Dirar Homouz

**Affiliations:** 1 Department of Physics, Khalifa University of Science and Technology, Abu Dhabi, 127788 United Arab Emirates; 2 Department of Physics, University of Houston, Houston, 77030-5005 TX USA; 3 Center for Theoretical Biological Physics, Rice University, Houston, 77030-1402 TX USA

**Keywords:** Carbon nanotube, Water dynamics, Hydrogen bond dynamics, Molecular dynamics simulations, Surfaces, interfaces and thin films, Structure of solids and liquids, Carbon nanotubes and fullerenes

## Abstract

Water dynamics inside hydrophobic confinement, such as carbon nanotubes (CNTs), has garnered significant attention, focusing on water diffusion. However, a crucial aspect remains unexplored - the influence of confinement size on water ordering and intrinsic hydrogen bond dynamics. To address this gap, we conducted extensive molecular dynamics simulations to investigate local ordering and intrinsic hydrogen bond dynamics of water molecules within CNTs of various sizes (length:20 nm, diameters: 1.0 nm to 5.0 nm) over a wide range of temperatures (260K, 280K, 300K, and 320K). A striking observation emerged: in smaller CNTs, water molecules adopt an icy structure near tube walls while maintaining liquid state towards the center. Notably, water behavior within a 2.0 nm CNT stands out as an anomaly, distinct from other CNT sizes considered in this study. This anomaly was explained through the formation of water layers inside CNTs. The hydrogen bond correlation function of water within CNTs decayed more slowly than bulk water, with an increasing rate as CNT diameter increased. In smaller CNTs, water molecules hold onto their hydrogen bond longer than larger ones. Interestingly, in larger CNTs, the innermost layer’s hydrogen bond lasts a shorter time compared to the other layers, and this changes with temperature.

## Introduction

Water confined inside the Carbon Nanotubes (CNTs) exhibits distinctive properties in terms of its dynamics and structure, significantly differ from the bulk water^1–3^. These unique properties render nanochannel highly promising for a wide array of applications, including drug delivery^4^, cancer treatment^5^, intercellular transport^6^, and desalination processes^7,8^. Consequently, there has been a considerable amount of research that has been performed to investigate the diffusion of water molecules within CNT. This topic has garnered substantial attention both in theoretical^9^ and experimental studies^10^. Such studies extracted valuable insight including an understanding of the temperature dependence of water diffusion within the nano-confinements. These insights may pave the way for the new design of synthetic nanochannels to mimic the performance of natural biological water channels.

Water serves as an ideal solvent for probing the effect of confinement, and studying the dynamics of hydrogen bonds of water confined within the CNTs offers valuable insight that extends to the other hydrogen bonding molecules. Theoretical studies of equilibrium properties of water inside CNTs have predominately relied upon molecular dynamics (MD) simulations^11–14^. Marti and coworkers^15^ were the first to report that the average number of hydrogen bonds of water molecules within CNT is less compared to the bulk water. Hummer et al.^16^ demonstrated that water molecules inside CNTs with diameters ranging from 6.76 to 8.11 Å arranged themselves as a single-file water chain. Marnon et al.^17^ found that the diffusivity of water within CNTs is lower than that of bulk water. However, Gordillo and coworkers^18^ found that the self-diffusion of water molecules within CNTs, along the tube axis direction, is faster than in bulk water. Recently^19–22^, we explored water dynamics within CNTs of various sizes and temperatures, utilizing both two-dimensional NMR diffusion relaxation (D-$T_{2eff}$) spectroscopy and MD simulations. Our result revealed that water molecules are inside two or more tubular components, each exhibiting a distinct self-diffusion coefficient. However, why the different tubular component exhibits different self-diffusion coefficients still remains elusive. Moreover, confined water within zigzag CNTs displayed multiple phase structures and transitions^23^. Noon et al.^24^ observed that altering the CNT diameter results in nanoconfined water manifesting a critical point, where liquid and solid phases coexist.

Confinement distinctly influences the inherent dynamics of hydrogen bonds (HBs) and the orientation of water molecule dipoles. Despite its importance, there’s a notable gap in research exploring the hydrogen bond dynamics of water within CNTs. Specifically, there’s a limited investigation into how variables like size and temperature impact these dynamics. Experimentally, HB dynamics have been studied using the femtosecond dynamics technique^25^, while theoretical studies have utilized MD simulations^26–28^. One fundamental measure of HB dynamics is the HB lifetime. Paul and Chandra^29^ studied HB dynamics at vapor-water and metal-water interfaces and revealed that the relaxation of HBs in these systems was slower compared to bulk water. Interestingly, they observed that inter-regional HB dynamics were faster compared to bulk water^29^. Examining the dynamics of hydrogen bonds provides valuable insights into the fundamental mechanisms that account for the accelerated motion of water molecules within CNTs.

Modeling water, despite its apparent simplicity as a liquid, presents complexity due to the intricate interplay between hydrogen bonding and van der Waals interactions. Due to the anomalous properties of water, no single water model can reproduce the physical properties of water over the whole range of thermodynamic states^30,31^. Li et al.^32^ reported that different water models can impact simulation results in confinement environments. Kumar et al.^33^ computed the structural and thermodynamic properties of water molecules confined inside the CNTs using the five different water models: TIP3P, modified TIP3P, SPC/E, SPCw (flexible water model), and POL3 (polorized watr model. They found the SPC/E water model is the optimum choice for the study of water confined inside CNTs considering both accuracy and computaional cost. Our work differs from most published studies that typically use density profiles to examine confined water. Instead, we employed a variety of parameters (radial distribution function, hydrogen bonds and their life times, density maps, orientational tetrahedral order ($S_q$) and translational tetrahedral Order ($S_k$) ) and established connections among these parameters to understand their impact on the water structure within CNTs. These metrics offer a distinct advantage over density profiles by providing deeper insights into the structural order of water confined within the hydrophobic spaces of CNTs.

## Methods

**Figure 1:**
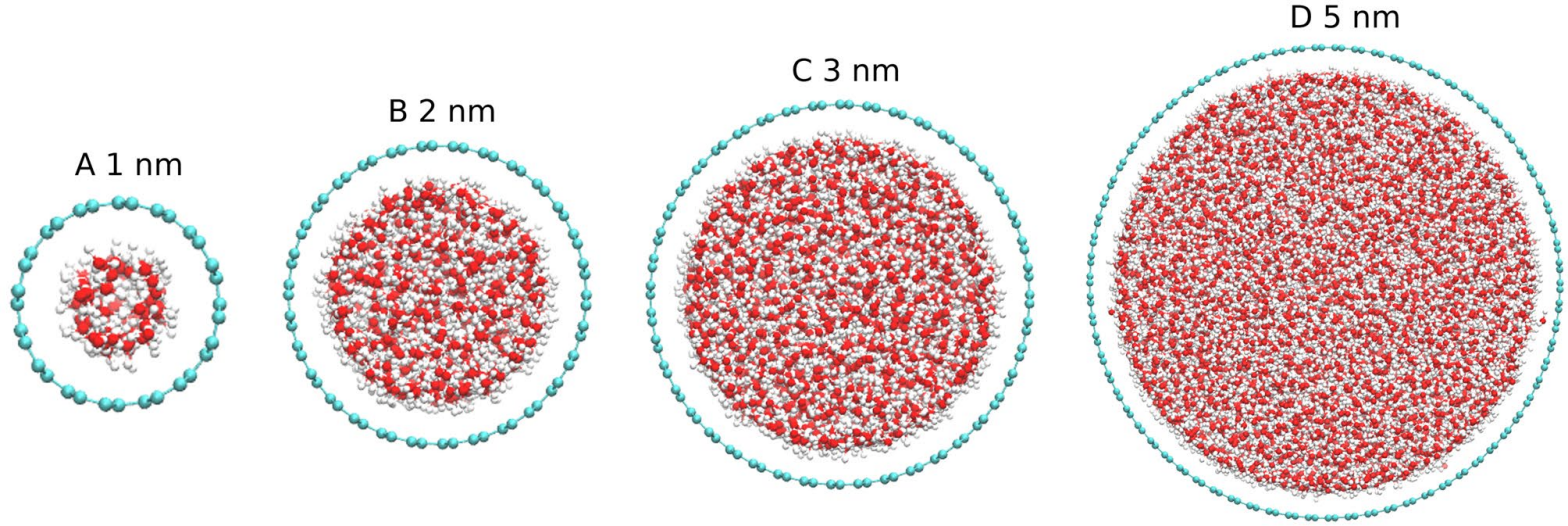
Schematic representation of Water-CNT used in the MD simulations: (**A**) 1.0 nm (8, 8), (**B**) 2.0 nm (15,15), (**C**) 3.0 nm (22,22), and (**D**) 5.0 nm (37, 37) of CNT filled with the water molecules. Oxygen, Hydrogen, and Carbon atoms are shown in red, white, and green colors. The chirality index of each arm-chair CNT diameter is given in brackets.

MD simulations were performed under a constant number of particles, pressure, and temperature ensemble to explore the ordering and the HB dynamics of water confined within CNTs of varying sizes. Water molecules were confined within arm-chair CNTs with diameters of 1.0 nm, 1.4 nm, 2.0 nm, 3.0 nm, and 5.0 nm, respectively. The chirality indices correspond to diameters are (8,8), (10,10), (15,15), (22,22), and (37,37), respectively. The initial configuration of the water-CNT system is depicted in Fig. 1. Notably, the length of the CNT was set to 20 nm for these simulations.

### Simulation setup

MD simulations were performed using NAMD package^34^, employing the SPC/E water model^35^, known for accurately predicting various bulk water properties. Non-bonded interactions between carbon atoms were described using the Lennard-Jones potential with parameters from Werder et al.^36^. CNT remained fixed throughout the simulations, imposing a positional restraint on each carbon atom. The system was immersed in a periodic simulation box of 4 $\times$ 4 $\times$ 23 $\text {nm}^3$. The dimensions of the simulation box vary along X and Y directions with the CNT diameter. The confined water inside the CNT was investigated in the temperature range of 260 K to 320 K, with increments of 20 K. The Langevin thermostat set the temperature to the desired value and a Nose-Hoover Langevin piston maintained the pressure at 1 bar, with a period of 100 fs and a damping time scale of 50 fs. Unlike previous studies with infinitely long CNTs along the axis, our simulation allowed water molecules to flow in and out of CNTs with a finite length of 20 nm, which gives a closer representative of real samples of CNTs used in various experiments. The Particle Mesh Ewald summation method (PME) was used to compute long-range electrostatic interactions. A time step of 2 fs is used for the integration of the equation of motion. Bonded interactions were computed at every time step, while non-bonded interaction was calculated every two steps, with a cutoff of 12 Å and a switching function of 10 Å. All the simulated systems were minimized for 10000 steps, followed by equilibration at the target temperature for 50,000 steps (100 ps) before the 50 ns production run. The system configuration was saved every 500 steps (1 ps) for subsequent analysis.

### Local order parameters for water

To characterize the arrangement of water molecules confined inside various sizes of CNTs, we have computed the order parameters that describe the water structure locally. Typically, confined water structures are mostly investigated using molecules density profile. In this work, we also used order parameters: Orientational Tetrahedral Order ($S_q$) and Transnational Tetrahedral Order ($S_k$). These parameters offer a distinct advantage over density profiles as they provide deeper insights into the structural order of water within the hydrophobic confinements of CNTs. The $S_q$ order parameter is given as:

1 $$\begin{aligned} S_q = 1 - \frac{3}{8} \sum _{i=1}^3 \sum _{j=i+1}^4 \left(cos\psi _{ij}+\frac{1}{3}\right)^2, \end{aligned}$$

$\psi _{ij}$, is the angle between lines joining the oxygen atom of the water molecule under consideration and its nearest neighbor oxygen atoms *i* and *j*. The angle between one atom to the four nearest neighboring atoms is measured and averaged. $S_q$ equals to 1 when $cos \psi _{ij}$ =-$\frac{1}{3}$, which is a regular tetrahedron. $S_q$ is widely used to describe the structure of liquid water, measuring the degree of flexibility in the arrangement of water molecules. $S_q$ value of 0 corresponds to an ideal gas, while a value of 1 corresponds to the regular tetrahedral structure of ice.

The $S_k$ order parameter is described as:

2 $$\begin{aligned} S_k = 1 - \frac{1}{3} \sum _{i=1}^4 \frac{(r_k - {\bar{r}})^2}{4{\bar{r}}^2}, \end{aligned}$$

$r_k$ is the distance between the oxygen atom in water molecules and its nearest neighbor of oxygen atoms. ${{\bar{r}}}$ is the average distance with the four nearest neighbor oxygen atoms. $S_k$ relies on the variance of radial distances between the oxygen atom of a water molecule under consideration with four nearest oxygen atoms. $S_k$ equals 1 corresponds to the regular tetrahedron. Water molecules near the CNT walls are excluded from the order parameter calculation due to their proximity to carbon atoms, which prevents them from occupying the center of a tetrahedral configuration. order parameters were calculated over a whole trajectory length i.e. 50 ns. Error bars represented by the standard deviations were estimated by calculating the order parameter for every 5.0 ns at a sampling rate of 1.0 ps.

### Hydrogen bond correlation function

The dynamic and thermodynamic characteristics of water are intricately connected to the nature of hydrogen bonding. Therefore, it is essential to gain a thorough understanding of the behavior of HBs in water molecules at various temperatures and within CNTs of different sizes. In order to investigate the dynamic processes involved in forming and breaking the HB network in a confined environment, we have calculated the HB auto-correlation function (HBACF) using the following geometrical criterion:

3 $$\begin{aligned} C_{HB}(t) = \frac{<b(t+\tau ) \cdot b(\tau )>}{<b(\tau )^2>} \end{aligned}$$

where $b(\tau )$ is the HB order parameter. For $b(\tau )$, a value of 1 is assigned if the two assigned water molecules form an HB at time $\tau$, and 0 if no HB is formed. The decay of HBACF is relatively swift, typically occurring within a few picoseconds, much faster compared to other auto-correlation functions for other simple liquids. The HBACF provides a measure of the likelihood that a pair of water molecules, initially bonded at time $\tau$, will remain bonded at time $t+\tau$. In other words, the HBACF provides insight into how persistent and dynamic the HB network in liquid environments. The geometric criteria for hydrogen bond selections are the following:

$$\begin{aligned} \theta \le 30^0 \\ |\mathbf{d_{OO}}| \le 3.50 \mathring{\textrm{A}} \end{aligned}$$

where $\theta$ is the OH$\cdot \cdot \cdot$O angle and $|\mathbf{d_{OO}}|$ is the distance between two oxygen atoms.

A bi-exponential function has been fitted in HBACF to obtain the parameters corresponding to the short-time ($\tau _1$) and long-time ($\tau _2$) decay components of the autocorrelation function.

4 $$\begin{aligned} C_{HB}(t) \cong A_1 e^{\left(\frac{-t}{\tau _1}\right)} + (1 -A_1) e^{\left(\frac{-t}{\tau _2}\right)} \end{aligned}$$

Finally, the lifetime of the hydrogen bond is calculated using Eq. (3):

5 $$\begin{aligned} \tau _{HB}(t) \cong A_1 \tau _1 + (1-A_1) \tau _2 \end{aligned}$$

where $\tau _{HB}$ is hydrogen bond lifetime. The last 20 ns trajectory was used for HBACF calculations.

## Results and discussion

**Figure 2:**
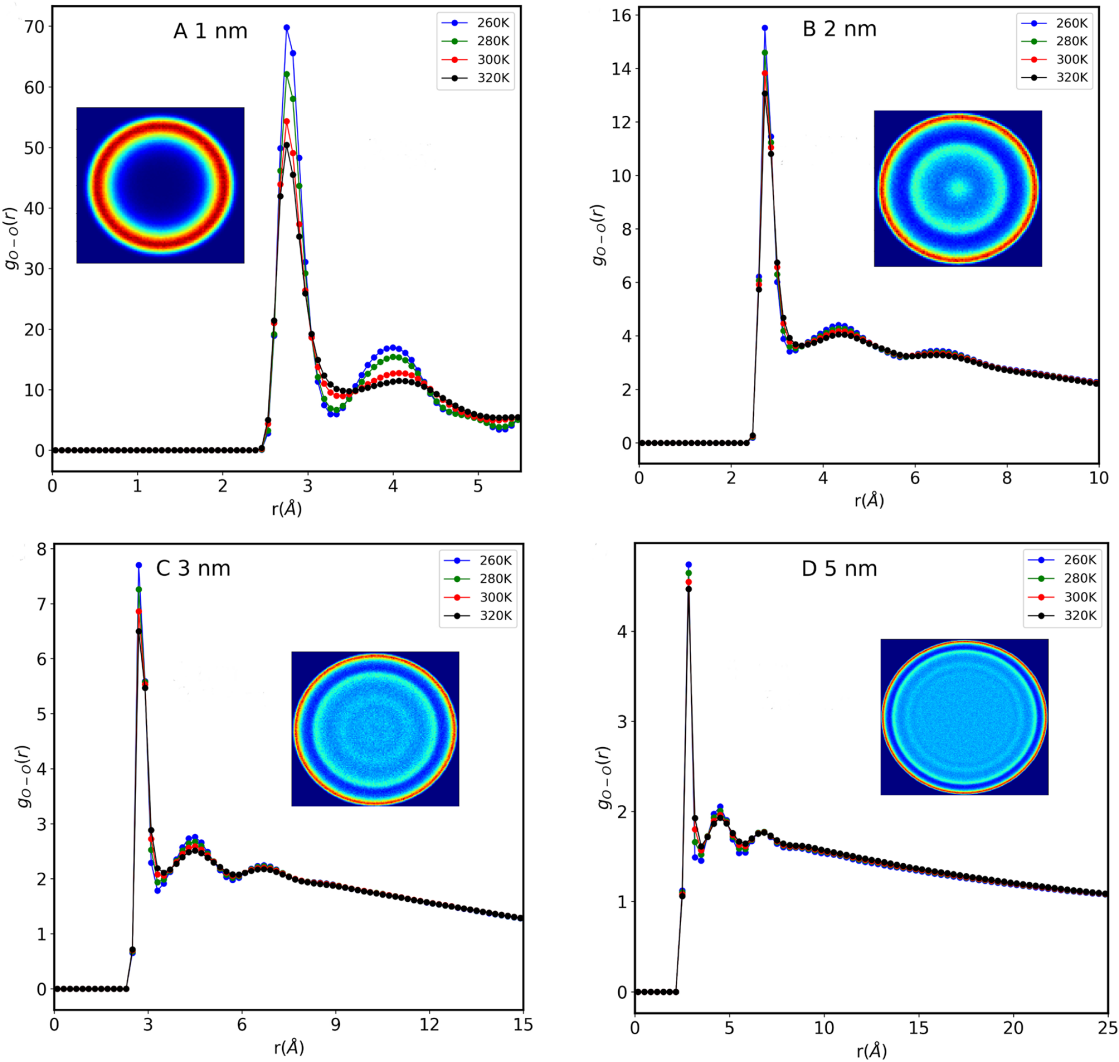
Radial distribution function of water Oxygen atoms inside various CNT sizes, at different temperatures (**A**) 1.0 nm, (**B**) 2.0 nm, (**C**) 3.0 nm and (**D**) 5.0 nm. The insets are the density map of water molecules in these CNTs, at 280 K, showing the formation of the layered structure. The high-water-density regions are shown in red color, and the low-density regions are shown in dark blue color.

It is well-known that confinement has a notable impact on water dynamics due to the change in HB structure of the molecules. In this work, we studied the effect of hydrophobic confinement on water dynamics inside different CNT sizes over a wide range of temperatures. We have computed the water radial distribution function (RDF) which is widely used, demonstrating the probable density of certain atoms as a function of distance. RDF is described by the probability of finding a particle at a distance *r* from a reference particle compared to the inhomogeneous distributions of the particle. The RDF for the oxygen atoms of the water molecules with respect to the CNT axis was calculated and shown in Fig. 2. The RDF has been computed using the following equation:

6 $$\begin{aligned} g_{O-O} (r) = \frac{1}{\rho ^2} \sum _{i=1}^N \sum _{j \ne i}^N <\delta (r+r_i-r_j)>, \end{aligned}$$

where $\rho$ is the number density. $r_i$ and $r_j$ represents the position of the $i^{th}$ and $j^{th}$ oxygen atoms of water molecule.

Figure 2 shows the RDF of confined water inside 1.0 nm, 2.0 nm, 3.0 nm, and 5.0 nm CNTs at different temperatures. Although the calculated results show slight variations among the different CNT sizes, the primary characteristic of the RDF curve remains consistent. The first peak corresponds to the first neighbor distance of the water molecule oxygen atom $\sim$ 2.84 Å. The amplitude of other peaks decreases rapidly with an increase in the distance between the water molecules and remains visible up to the third layer. The density map of confined water molecules, shown in the insets of the figure, reveals a layered structure, consistent with results reported from both experimental studies and molecular dynamics (MD) simulations.^9,20–22^. Furthermore, these results show that the ordering of water molecules is inversely proportional to temperature; the intensities of the RDF peaks decrease upon increasing the temperature. We find that the first peak position does not change with the increase in confinement size, however, the second peak shifts slightly with the increase in confinement size. The decrease in RDF peaks indicates a transition from the long-range structural order to a liquid-like phase. In addition, we also calculated the density map of oxygen atoms of water molecules in the XY plane. The density map was obtained by dividing the corresponding plane into the square bin of 0.1 Å length and then counting the number of oxygen atoms in each square bin. Higher oxygen densities are shown in red, while the low densities are shown in blue color. The inset of Fig. 2 shows the density map of water in each CNT at a temperature of 280 K. In all cases, density maps of water molecules are arranged in layered structures (rings in the density map), in agreement with the previous work^9,20,21^. The density map displays that upon increasing the CNT sizes the number of layers increases, consistent with RDF results. This layered structure observed in our simulated system does not depend on the temperature. It is clear now that confinement forces water molecules to form a layered structure but how the water ordering varies in these layered structures has not been discussed previously. Recently, both with experimental results and MD simulation, we found that water molecules close to the center of CNT diffuse faster compared to the water molecules adjacent to the CNT wall, but the exact explanation of this observation remains elusive. To further address this, we have computed the local water order parameter. We relied on two widely used order parameters for liquids^37^ i.e. Orientational tetrahedral and Translational tetrahedral order parameters.

**Figure 3:**
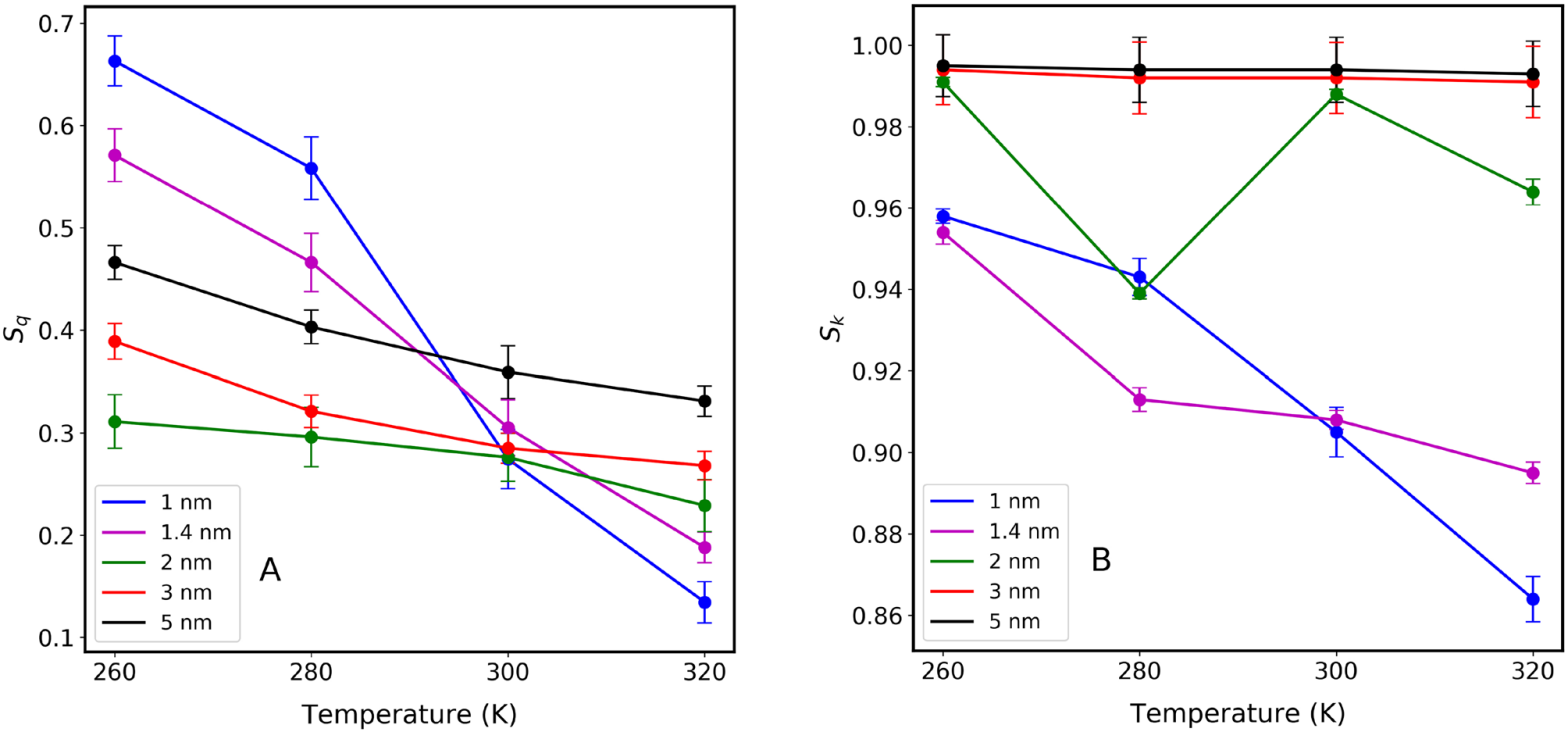
(**A**) Orientational tetrahedral order parameter ($S_q$) of confined water molecules inside various CNT sizes, at different temperatures, (**B**) Translational tetrahedral order parameter ($S_k$) of confined water molecules inside various CNT sizes, at different temperatures. $S_q$ and $S_k$ are obtained by considering the total number of water molecules within the CNT channels. Error bars indicate the standard deviations of the both parameters.

**Figure 4:**
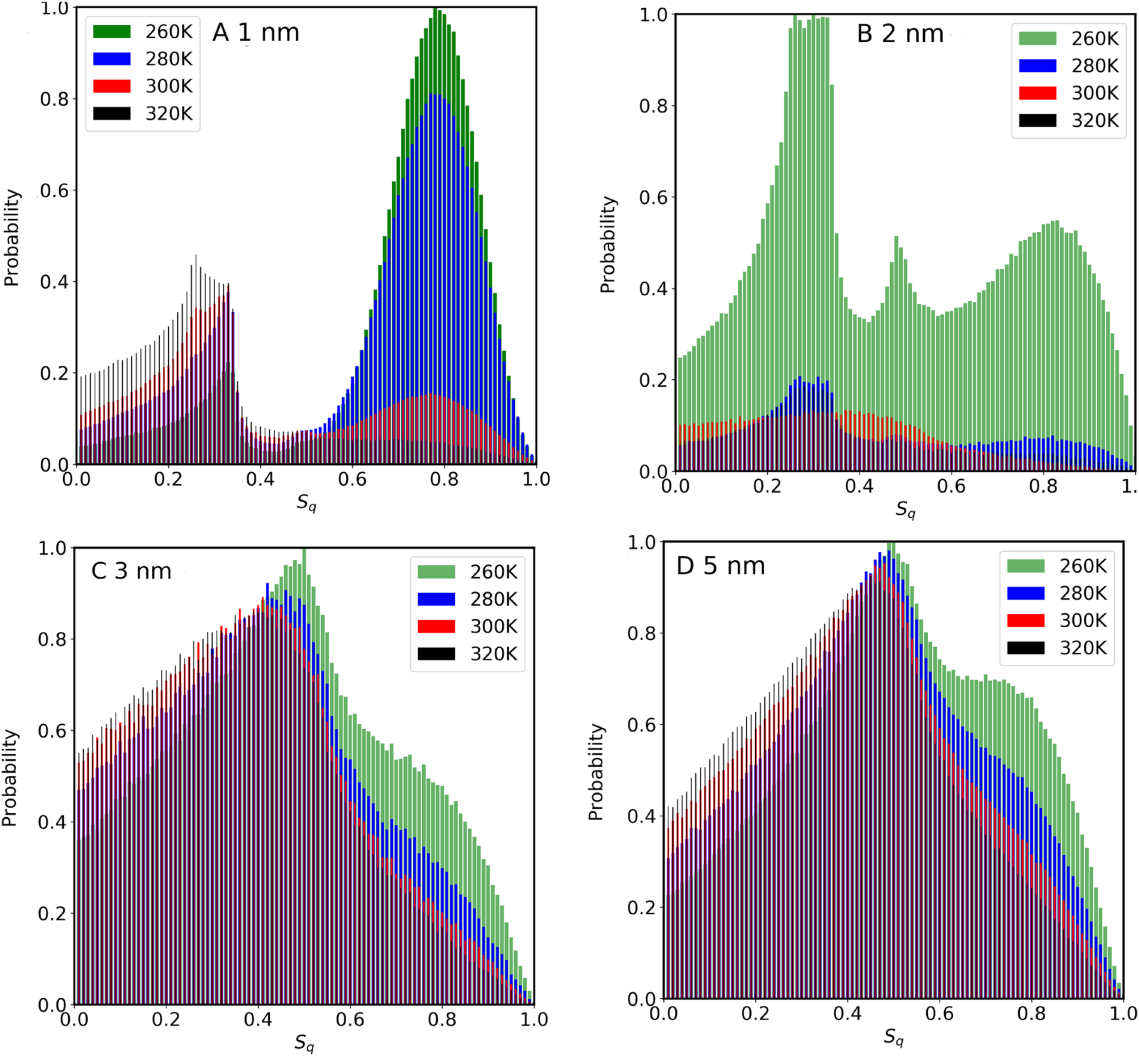
Probability distribution of Orientational tetrahedral order parameter of confined water molecules inside various CNT sizes, at different temperatures (**A**) 1.0 nm, (**B**) 2.0 nm, (**C**) 3.0 nm and (**D**) 5.0 nm. The analysis takes into account the total number of water molecules confined within the CNT channel.

The orientational tetrahedral order parameter $S_q$ has been calculated for water inside different sizes of CNTs (Fig. 3A). As seen from Fig. 3A, $S_q$ in each CNT size is inversely proportional to temperature. This is expected since thermal energy provided to water molecules changes the structure from a quasi-icy structure toward a gas phase. At 300 K, the $S_q$ for the confined water in 1.0 nm CNT is 0.2744, 0.305 for 1.4 nm, 0.276 for 2 nm, 0.285 for 3 nm, and 0.359 for 5 nm. The results indicate that confined water molecules behave differently compared to the bulk water^37^. Results, excluding those in 2.0 nm indicate that $S_q$ is also inversely proportional to the CNT size. At first glance, this might be explained by the effect of the CNT wall on water molecules, at smaller CNTs compared to those in larger CNT sizes. It is important to point out that the analysis of Fig. 3A is based on considering the total number of water molecules inside CNTs. In Fig. 4, we observe the probability distribution of $S_q$ parameters versus temperature for all the CNT sizes considered in this study including the total number of water molecules. The distribution of this parameter for water molecules inside 1.0 nm and 2.0 nm shows two distribution peaks. The distribution in the case of 2.0 nm is shifted toward a low-value gas phase causing the overall weighted value of $S_q$ in 2.0 nm to give a smaller value compared to that of 1.0 nm as seen before (Fig. 3A). At larger CNT size the distribution gets broader and the overall value of $S_q$ decreases as also seen in Fig. 3A.

In Fig. 3B, the average translational tetrahedral order parameter ($S_k$) of water molecules inside CNTs of various sizes is presented at different temperatures. Specifically, at 300 K, the $S_k$ for a 1.0 nm CNT is 0.905. As the CNT size increases, the $S_k$ values exhibit an upward trend: 0.988 for 2.0 nm, 0.992 for 3.0 nm, and 0.994 for 5.0 nm. We find that by increasing the confinement size, the $S_k$ value tends to approach the bulk value of 0.999, as reported in previous studies. This is because more water molecules fill the empty space, leading to a density approaching that of the bulk. Similar trends were observed in RDFs. Unlike $S_q$, $S_k$ does not seem to offer a useful complementary measure of the change in the local structure of water molecules inside CNTs^37^. Since $S_k$ focuses on the variance of radial distances between an oxygen atom and the four nearest oxygen atoms, therefore, for 1.0 nm CNT, the $S_k$ value decreases with an increase in temperature. This is because at 260 K, the water molecules are ordered, radial distance fluctuations are less, and as the temperature is increased water molecules fluctuate more. Conversely, for the 2.0 nm CNT, the $S_k$ value decreases initially with temperature, but further temperature increments result in an increase. Notably, for 3.0 nm and 5.0 nm CNTs, the $S_k$ value shows minimal variation with temperature changes.

**Figure 5:**
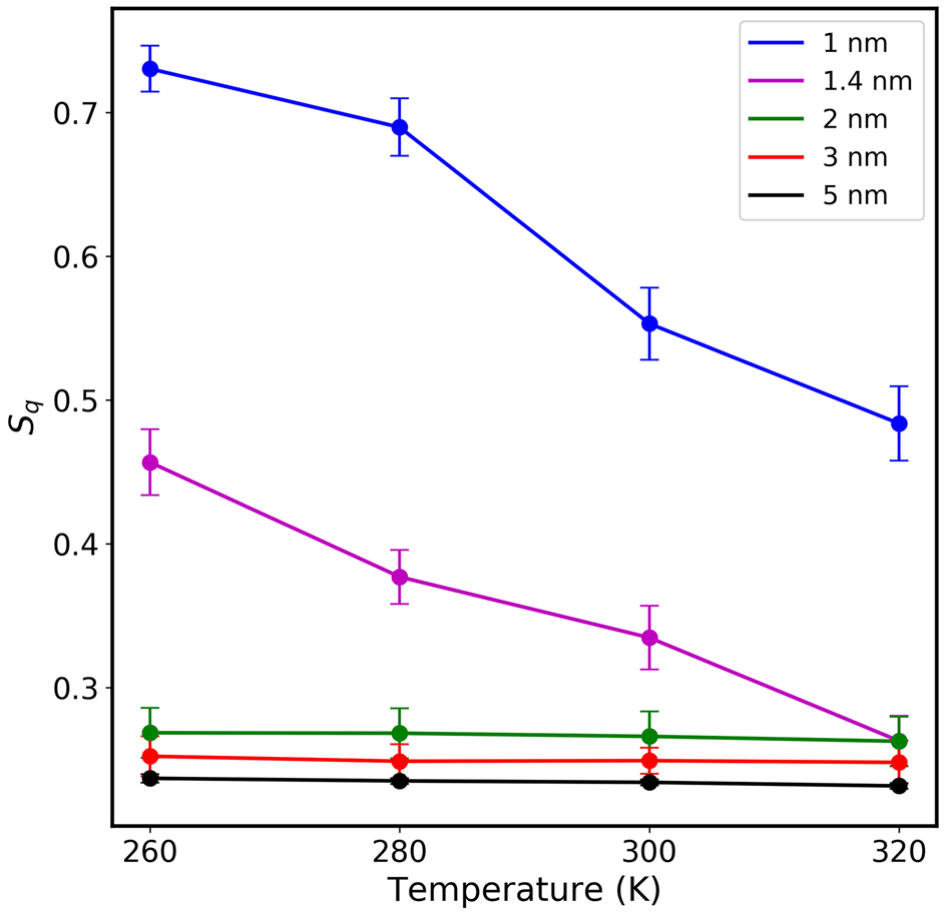
Orientational tetrahedral order parameter ($S_q$) of confined water molecules inside various CNT sizes, at different temperatures. To exclude the effect of the CNT walls, the analysis considers water molecules within a cylindrical shell ranging from 0 Å to 3 Å, with 0 Å referring to the center of CNT channel.Error bars represent the standard deviations of $S_q$.

**Figure 6:**
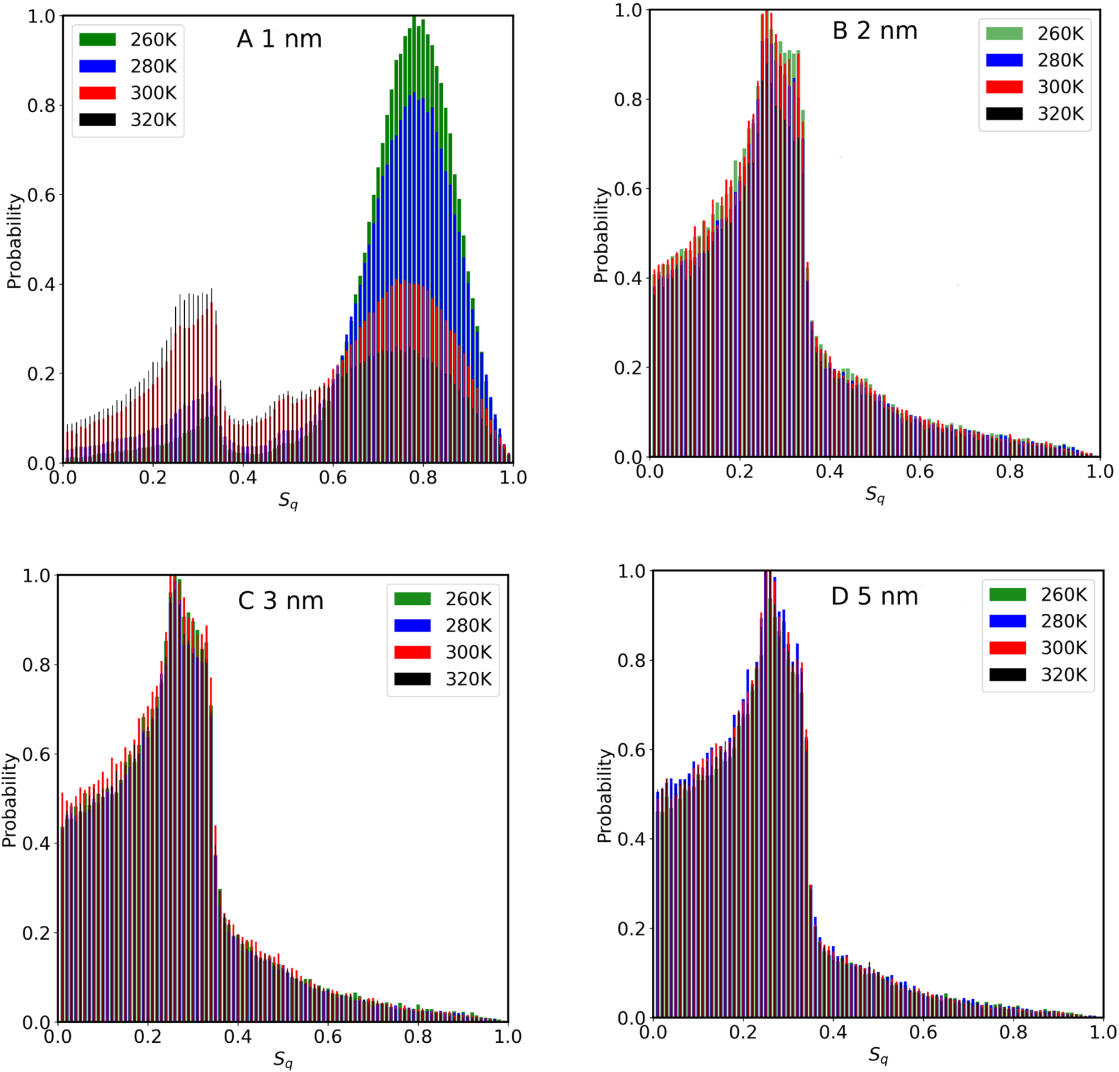
Probability distribution of Orientational tetrahedral order parameter of confined water molecules inside various CNT sizes, at different temperatures (**A**) 1.0 nm, (**B**) 2.0 nm, (**C**) 3.0 nm and (**D**) 5.0 nm. Water molecules inside a cylindrical shell ranging from 0 Å to 3 Å are considered in this analysis. Note the difference between the results presented here and those in Fig. 4, where the total number of water molecules was considered. This distinction is particularly evident in the data for water confined within the 2.0 nm CNT.

Previously, we reported^10,20–22^, as well as other research groups^9^ that water molecules inside CNT sizes rearrange themselves in coaxial cylindrical shapes, diffusing with different motilities along the axis of the CNTs. The self-diffusion coefficients versus temperature increase upon increasing CNT size, reaching maximum value when CNT size is about 2.0 nm - 2.5 nm, then decrease and reach that of bulk phase at larger CNT sizes ( > 4.0 nm). The anomalous behavior of $S_q$ in the data of Fig. 3A at 2.0 nm might be related to water mobilization. To further investigate this, we have divided the volume inside each CNT channel into several regions, and then $S_q$ values are calculated for each of those regions. For water molecules located within a cylindrical shell extending from the center of the CNT to 3.0 Å, results are presented in Fig. 5. Unlike the results presented in Fig. 3 (where the total number of water molecules was considered), the anomalous behavior observed inside 2.0 nm is no longer seen, since the CNT wall effect has been excluded. At each temperature, the $S_q$ is inversely proportional to the CNT size (including 2.0 nm data). The probability distribution $S_q$ of the water molecules from the center of the CNT to 3.0 Å (shown in Fig. 6) reflects this fact. Notice the difference between the results of 2.0 nm in this case compared to that of Fig. 4 where all the water molecules were considered for the calculation. This comparison confirms that inside 2.0 nm CNT size, water molecules close to the walls have a more structured quasi-icy phase compared to those at the center of the channels. This is further supported by observing the probability distribution of $S_q$ for water within larger CNT sizes. With ample space available, the structural arrangement of water molecules tends to lean towards a gas phase (lower values of $S_q$).

To elucidate how the local ordering of water molecules affects the mobility of water molecules and hydrogen bonds, we have calculated both the self-diffusion coefficient ($D_z$) and HBs at 300K. At this temperature, the $D_z$ value of the confined water in the 1 nm CNT is 0.882 $\times$ $10^{-5}$ $cm^2/s$. As the CNT size increases, the $D_z$ values increase 2.33 $\times$ $10^{-5}$ $cm^2/s$ in 2nm CNT, 2.74 $\times$ $10^{-5}$ $cm^2/s$ in 3 nm, and 2.42 $\times$ $10^{-5}$ $cm^2/s$ in 5 nm, while the number of HB is 2.22 for water in 1 nm CNT, 2.61 in 2 nm, 2.71 i 3nm, and 2.80 in 5nm CNT. The result indicates that order parameters are directly proportional to the self-diffusion coefficient and HBs.

As the RDF results and density map show that water molecules inside the CNT channel of different diameters are arranged in a layered structure, it would be important to examine the dynamics of water in these layers. We have calculated the HBs for water molecules in each layers . Figure S1 shows the average number of hydrogen bonds per water molecule in various water layers inside differnet CNTs sizes.

### Hydrogen bond dynamics

**Figure 7:**
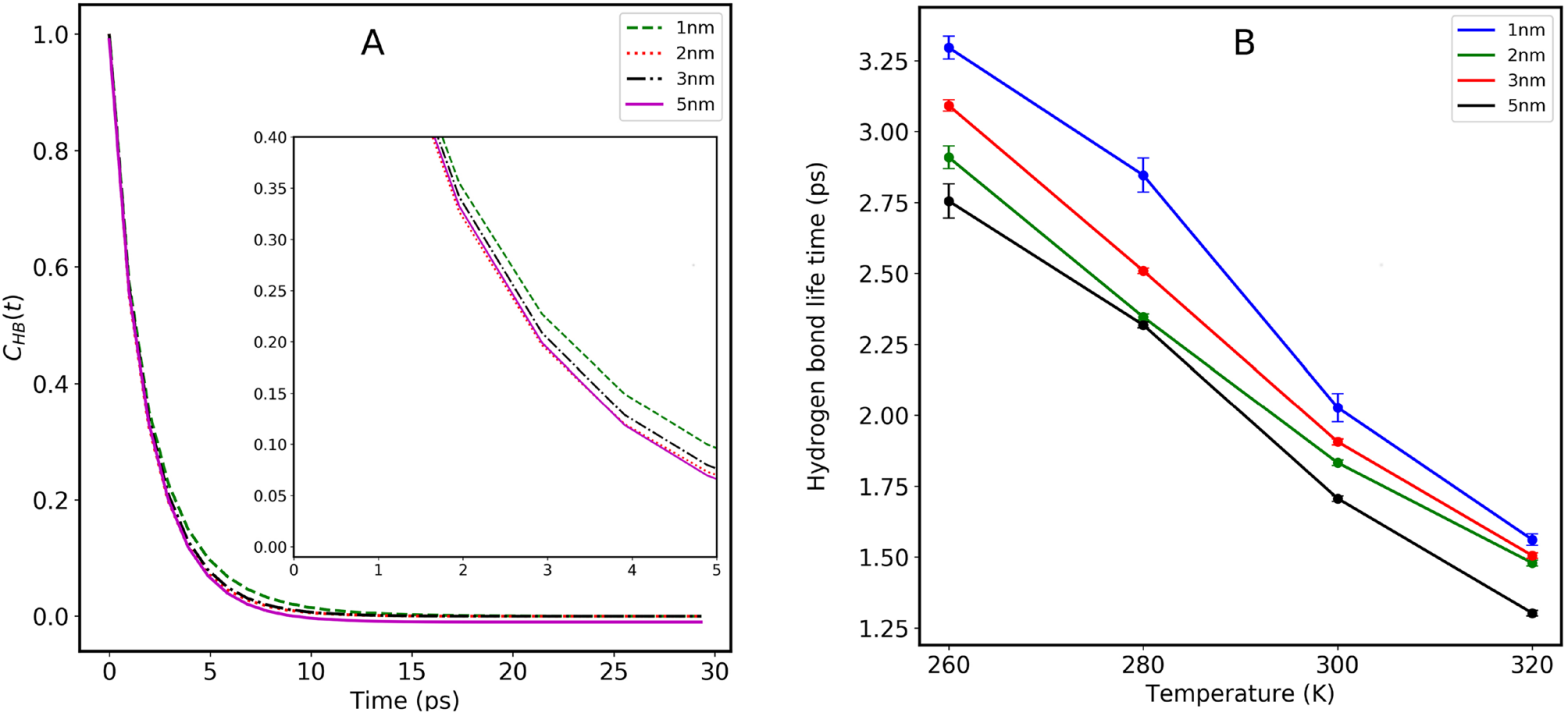
(**A**) Hydrogen bond auto-correlation function of confined water molecules inside various CNT sizes, at 300 K. The inset is the magnification of the data between 0 to 5 ps. (**B**) Temperature dependence of the hydrogen bond lifetime of the confined water molecules, at different temperatures.

To evaluate the resilience of HBs among water molecules within the CNTs, we analyzed their longevity. This assessment included computing the HBACF, discussed in the method section, to investigate how both the size of confinement and temperature affect the stability of these crucial bonds. The total number of water molecules within the CNTs was considered. Figure 7 illustrates the variation of HBACF for different CNT sizes (1.0 nm, 2.0 nm, 3.0 nm, and 5.0 nm) at room temperatures. In Fig. 7’s left panel, we observe an exponential decline in HBACF over simulation time across all CNT sizes. However, the rate of this decline noticeably varies with different confinement sizes. The inset within the figure distinctly highlights that water molecules confined within smaller CNTs (1.0 nm) sustain their HB networks for extended periods compared to those within larger CNTs.

The HB lifetime was determined by fitting the double exponential function as detailed in the method section. Figure 7B illustrates the HB lifetime across different CNT sizes at various temperatures, revealing a decreasing trend with rising temperature as well as CNT size. At 260K for smaller CNT (1nm), the HB lifetime is $\sim$ 3.3 indicating an ice-like structure which is consistent with our previous observation. In our previous study^22^, we calculated the HB lifetime using the TIP3P water model. At 300 K, the HB lifetime values for different CNT sizes were 1.0 ps (in 1.0 nm), 0.96 ps (in 2.0 nm), 0.94 ps (in 3.0 nm), and 0.91 ps (in 5.0 nm CNT). These values are notably smaller compared to the SPC/E water model. This shows that the HB network is less structured in the TIP3P model, emphasizing that the SPC/E water model is the optimal choice for the study of water dynamics.

As we know water molecules inside CNT are arranged in coaxial water cylinerical (CWC) sheets^20–22^. Therefore, we examine the HB dynamics in each CWC sheet using the HB lifetime. Table 1 presents the hydrogen bond information for water molecules corresponding to each CWC sheet inside different CNT sizes at temperatures 260 K, 280 K, 300 K, and 320 K. The table highlights the notable differences in water dynamics among the various CWC sheets. According to the data, at low temperatures, the outermost layer of water exhibits the longest HB lifetime providing a potential explanation for its slower diffusion, whereas as we increase the temperature, the HB lifetime remains the same throughout the different layers. These findings align with those of the previous studies.

**Table 1:** The hydrogen bond lifetime of water molecules within different CNT sizes, at different temperatures. The Water shells in the table correspond to the coaxial water cylindrical sheets formed in CNTs due to water confinement.

CNT ( in nm)	Water-Shell (in Å)	Temperature (in K)			
		260K	280K	300K	320K
1.0	0-5	3.296	2.842	2.08	1.561
		(±0.05)	(± 0.06)	(±0.05)	(± 0.02)
	Outer-shell	3.09	2.646	1.84	1.482
	(5–10)	(±0.04)	(± 0.02)	(±0.01)	(± 0.01)
2.0	Inner-shell1	2.755	2.349	1.83	1.48
	(2–5)	(±0.04)	(± 0.02)	(±0.01)	(± 0.01)
	Inner-shell2	2.809	2.364	1.842	1.471
	(0–2)	(±0.14)	(± 0.08)	(±0.06)	(± 0.04)
	Outer-shell	3.13	2.594	1.903	1.510
	(10–15)	(±0.02)	(± 0.01)	(±0.01)	(± 0.01)
3.0	Inner-shell1	3.01	2.51	1.91	1.510
	(6–10)	(±0.02)	(± 0.01)	(±0.06)	(± 0.01)
	Inner-shell2	3.09	2.537	1.91	1.509
	(0–6)	(±0.03)	(± 0.02)	(±0.01)	(± 0.01)
	Outer-shell	2.785	2.51	1.69	1.504
	(20–25)	(±0.01)	(± 0.01)	(±0.01)	(± 0.01)
5.0	Inner-shell1	2.746	2.610	1.70	1.504
	(17–20)	(±0.04)	(± 0.01)	(±0.01)	(± 0.01)
	Inner-shell2	2.751	2.518	1.70	1.502
	(13–17)	(±0.03)	(± 0.01)	(±0.01)	(± 0.01)
	Inner-shell3	2.755	2.552	1.70	1.504
	(0–13)	(±0.01)	(± 0.02)	(±0.01)	(± 0.01)

## Conclusion

In this study, we performed MD simulations to examine how both size and temperature influence the ordering of water and the dynamics of hydrogen bonds within confined CNTs. Our analysis spanned pore sizes from 1.0 nm to 5.0 nm across temperatures ranging from 260 K to 320 K. We focused on water molecule distribution and structural ordering of water in these confined environments. Additionally, we explored the impact of temperature and confinement size on the hydrogen bonds and their lifetime. The radial distribution and density maps showed that water molecules forms cylindrical layers. To examine the ordering of watrer molecuels in these layers, we calculated the order parameters in various warter layers. The Orientational order parameter highlighted that in smaller nanotubes water adopts an ice-like structure near the tube walls, with the ordering pattern changing as the temperature rises.

We find that water near the CNT center displayed a lower value of $S_q$ ($\sim$ 0.34) compared to that of water molecules near the wall ($\sim$ 0.63). The higher $S_q$ value demonstrates that water molecules near the CNT wall form an ice-like structure. Due to this a significant reduction in the diffusion coefficient of water molecules close to the wall was observed both in experiments and MD simulations. Our findings for 2.0 nm CNT revealed the formation of an ice-like structure of water molecules adjacent to the CNT wall at low temperatures, a novel observation not previously reported. For 2.0 nm CNTs, the orientational order parameter ($S_q$) exhibited anomalous behavior with increasing temperature, consistent with diffusion observations from prior studies. The hydrogen bond correlation function of water within CNTs exhibited a slower decay compared to bulk water, with the decay rate decreasing as CNT diameter increased. In larger CNTs, the hydrogen bond lifetime of the innermost layer was shorter than that of other layers and was temperature-dependent. We also demonstrate the water ordering CNT size above 3.0 nm is almost the same with that in the bulk. This study provides insights into the behavior of water molecules within a hydrophobic confinement. Overall, our study offers a detailed understanding of how confinement sizes and varying temperatures impact the structure and dynamic properties of water. These findings are important for applications in nanofluidics and other applications where water-CNT interactions are crucial. The discovery of ice-like structures and the anomalous temperature-dependent behavior of order parameters in specific CNT sizes provide new insights into the unique properties of confined water.

### Supplementary Information

Supplementary Information.

## Data Availability

The data generated in the current study are available from the corresponding author upon reasonable request.
