# Supplementary material for: Dynamics of confined water inside carbon nanotubes based on studying tetrahedral order parameters
Source: Sci Rep. 2024 Jul 5;14:15480. doi: 10.1038/s41598-024-66317-1 (PMC11226439; doi:10.1038/s41598-024-66317-1)
Supplement: Supplementary file 1 — Supplementary Information. [file 41598_2024_66317_MOESM1_ESM.pdf]

Supplemental information for Dynamics of Confined Water inside Carbon Nanotubes Based on Studying Tetrahedral Order Parameters

Amit Srivastava[1], **Sufian Abedrabbo**[1], **Jamal Hassan**[1], **and Dirar Homouz**[1,2,3,*]

[1]Department of Physics, Khalifa University of Science and Technology, Abu Dhabi 127788, United Arab Emirates.
[2]Department of Physics, University of Houston, Houston, TX 77030-5005, USA.
[3]Center for Theoretical Biological Physics, Rice University, Houston, TX 77030-1402, USA
[*]dirar.homouz@ku.ac.ae

ABSTRACT

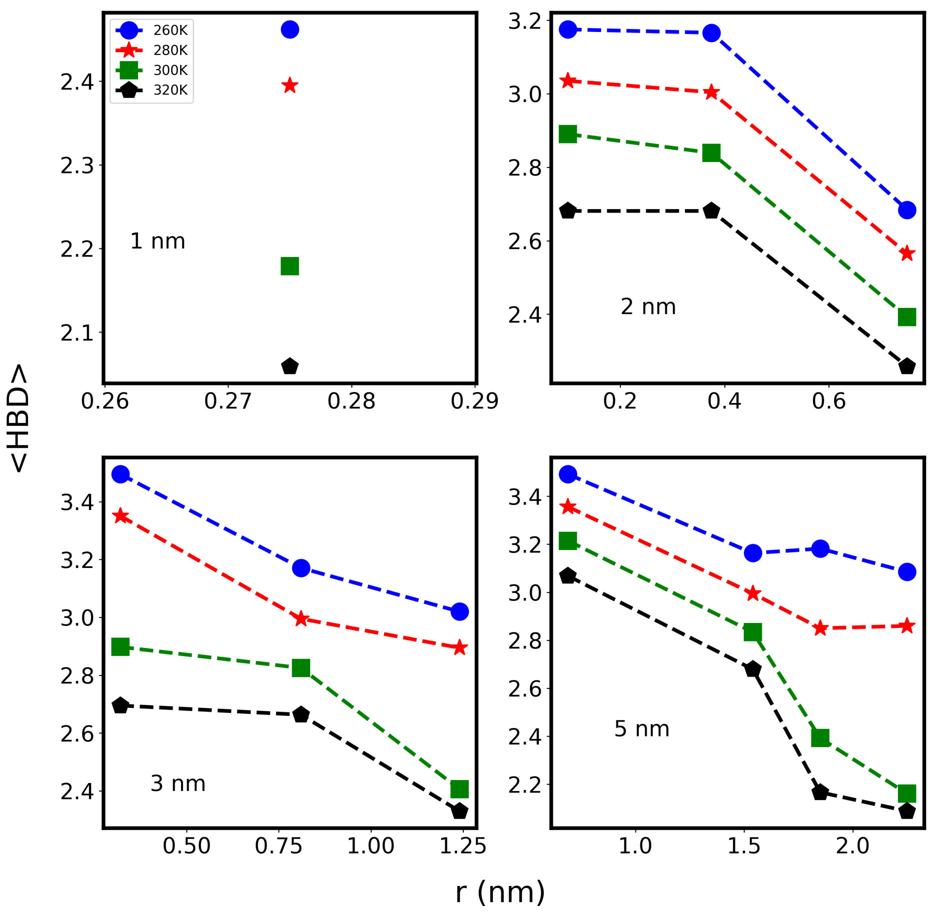

Fig. S1. Average number of hydrogen bonds per water molecule in various water layers inside different CNT sizes. The x-axis shows the distance of the chosen water layer from the center of CNTs. Blue, red, green, and black color data points correspond to temperatures 260 K, 280 K, 300 K, and 320 K, respectively
